# GnRH Antagonists with or without Add-Back Therapy: A New Alternative in the Management of Endometriosis?

**DOI:** 10.3390/ijms222111342

**Published:** 2021-10-20

**Authors:** Jacques Donnez, Marie-Madeleine Dolmans

**Affiliations:** 1 Société de Recherche pour l’Infertilité (SRI), 143 Avenue Grandchamp, B-1150 Brussels, Belgium; 2 Medical School, Université Catholique de Louvain, 1200 Brussels, Belgium; 3 Gynecology Research Unit, Institut de Recherche Expérimentale et Clinique, Université Catholique de Louvain, 1200 Brussels, Belgium; marie-madeleine.dolmans@uclouvain.be; 4 Gynecology Department, Cliniques Universitaires Saint Luc, 1200 Brussels, Belgium

**Keywords:** endometriosis, pelvic pain, dysmenorrhea, progesterone resistance, GnRH antagonist, add-back therapy, elagolix, relugolix, linzagolix

## Abstract

To evaluate the effectiveness of a new class of medical drugs, namely oral gonadotropin-releasing hormone (GnRH) antagonists, in the management of premenopausal women with endometriosis-associated pelvic pain. We reviewed the most relevant papers (*n* = 27) on the efficacy of new medical alternatives (oral GnRH antagonists) as therapy for endometriosis. We first briefly summarized the concept of progesterone resistance and established that oral contraceptives and progestogens work well in two-thirds of women suffering from endometriosis. Since clinical evidence shows that estrogens play a critical role in the pathogenesis of the disease, lowering their levels with oral GnRH antagonists may well prove effective, especially in women who fail to respond to progestogens. There is a need for reliable long-term oral treatment capable of managing endometriosis symptoms, taking into consideration both the main symptoms and phenotype of the disease. Published studies reviewed and discussed here confirm the efficacy of GnRH antagonists. There is a place for GnRH antagonists in the management of symptomatic endometriosis. Novel algorithms that take into account the different phenotypes are proposed.

## 1. Introduction

The aim of this review is to present, based on recent literature, data on a new class of medical drugs, namely oral gonadotropin-releasing hormone (GnRH) antagonists, for the management of symptomatic endometriosis, a common chronic inflammatory disease causing pain and infertility [1,2,3] and affecting between 5% and 10% of women of reproductive age [1,3]. Since the original publication by Nisolle and Donnez, it has been widely accepted that there are three distinct phenotypes of the disease (peritoneal, ovarian, and rectovaginal endometriosis) [2]. Estradiol (E2) has proinflammatory and antiapoptotic effects on endometrial and endometriotic cells and plays a crucial role in the pathogenesis of endometriosis [2,3]. Blocking ovulation and menstruation by means of hormone therapies may in theory be disease-modulating and control the symptoms of endometriosis [4]. Lowering E2 to within the 30–60 pg/mL range according to the threshold hypothesis [5] could be another approach offering the best compromise between efficacy and safety. The goal of this review is to evaluate the place of several oral GnRH antagonists in the management of symptomatic endometriosis.

A literature search was conducted through an electronic database (PubMed, Embase) up to April 2021. The following key words were entered: endometriosis, GnRH antagonist, and add-back therapy. From 2010 to 2021, 83 manuscripts reported data and results on GnRH antagonist medical therapy for endometriosis. The search was limited to peer-reviewed full texts in English, reporting data on medical treatment by GnRH antagonist. After identifying original articles and reviews that were methodologically sound, well written, updated, informative, and well balanced, and taking into account duplicated results and plagiarism, the authors selected and reviewed 27 relevant original papers on oral GnRH antagonists in the management of endometriosis in symptomatic premenopausal women.

## 2. Why Are Estroprogestins and Progestins Only Effective in Two-Thirds of Women?

Casper [6] strongly asserts that progestin-only pills constitute a better first-line approach than estroprogestins, but according to Vercellini et al. [7], progestin-only therapy should be reserved for women with contraindications or intolerance to estroprogestins. Use of oral contraceptive pills (OCPs) containing estroprogestins is considered off-label, despite being included in various guidelines. In one of their numerous reviews, Vercellini et al. (8–12) advocated the use of estroprogestins for the treatment of endometriosis, but 33% of patients given estroprogestins and/or progestins do not respond to therapy [6,7,8,9,10,11,12,13].

Only one randomized placebo-controlled clinical trial of OCPs in endometriosis has ever been published [14]. OCP administration resulted in around a 50% reduction in dysmenorrhea, but there was no beneficial effect of OCPs on non-menstrual pelvic pain or dyspareunia. Moreover, none of the studies reported data on their efficacy according to lesion phenotype. In a recent investigation of Cochrane reviews, Brown and Farquhar [15] concluded that while OCPs are widely used to treat endometriosis-related pain, evidence of their efficacy is limited.

Buggio et al. [16] conducted an excellent review of available progestins used in the management of endometriosis, including norethisterone acetate (NETA), dienogest, desogestrel, cyproterone acetate, depot medroxyprogesterone acetate (DMPA), and the levonorgestrel-releasing intrauterine system (LNG-IUS). According to these authors [16], all available progestins are equally effective at controlling pain symptoms in two-thirds of women with endometriosis, and there is no evidence to suggest the superiority of one progestin over another. They also concluded that NETA should be considered first-line treatment, given the favorable cost-effectiveness profile. A study comparing NETA and dienogest [17] reported that 70% and 72% of patients respectively were satisfied, while a substantial proportion of around 30% were left dissatisfied, 10% of whom were very dissatisfied. Moreover, Vercellini et al. [17] reported that in poor responders to OCPs, administration of dienogest was not associated with any statistically significant improvement in overall pain relief, psychological status, sexual functioning, or health-related quality of life.

Progesterone receptor (PR) deficiency causing progesterone resistance in endometriotic lesions shows the absence of response in more than 30% of women treated with progestins to be linked to survival of endometriotic tissue [18,19,20,21,22]. In 1997, Nisolle and Donnez [2] hypothesized that PRs were biologically inactive, suggesting the notion of progesterone resistance, later confirmed by Attia et al. [22]. Bulun et al. [23] demonstrated that in endometriotic implants, estrogen receptor alpha (ERα) action is reduced, but its beta activity (ERβ) is upregulated. This leads to loss of PRB, which is then unable to induce 17β-HSD2 and inactivate local E2, ultimately leading to high levels of E2 [22,23,24,25,26,27]. The origins of progesterone resistance in adult women were reviewed by Patel et al. [19], Bulun et al. [23,24,25,26], and Donnez and Dolmans [21]. Key causes include inflammation and oxidative stress, as very recently reviewed by Cacciottola et al. [28]. As emphasized by Donnez et al. [29] and Cacciottola et al. [28], red blood cells, apoptotic endometrial tissue, and endometrial debris transplanted into the peritoneal cavity by tubal reflux are potential inducers of oxidative stress [29,30,31,32,33,34].

## 3. Why Do We Need New Options?

New options are needed because of concerns about the effects of available drugs, namely:

- One-third of patients are non-responders to estroprogestins and progestin-only medication due to progesterone resistance [6,8,9,19,20,21,22,23,24,25,26].
- Among new drugs, selective progesterone receptor modulators (SPRMs) are not a viable option, as they also induce endometrial changes in ectopic foci [35,36,37]. Moreover, their use for fibroids is strictly limited to defined indications due to the possibility of triggering liver disease, while their use in endometriosis is off-label.
- GnRH agonists are effective at treating endometriosis symptoms, but have numerous limitations, including a delayed therapeutic impact because of the flare-up effect, suppression of E2 to less than 20 pg/mL, inability to titrate E2 levels, and unpredictable reversibility of treatment when injectable depot forms of GnRH agonists are used [38,39,40,41,42,43].

## 4. How Do We Achieve Partial E2 Suppression? Is Gnrh Antagonist the Best New Option?

In theory, the ideal solution would be to lower E2 levels enough to induce amenorrhea and treat symptoms, while maintaining sufficient E2 values to mitigate vasomotor menopausal symptoms (essentially hot flushes) and bone mineral density (BMD) loss. According to the threshold hypothesis proposed by Barbieri [5] several years ago, partial suppression of E2 to within the 30–60 pg/mL range could be the best available compromise between efficacy, tolerance, and safety [13]. As estrogens play a crucial role in survival and vascularization of endometriotic implants, it is entirely reasonable to consider lowering their concentrations as a therapeutic approach [13,21].

GnRH antagonists have recently been the focus of several papers [44,45,46,47,48,49,50,51,52,53,54,55,56,57,58,59,60,61,62,63]. These drugs cause competitive blockage of the GnRH receptor and thereby dose-dependently suppress production of follicle-stimulating hormone (FSH) and luteinizing hormone (LH), and inhibit secretion of ovarian steroid hormones without inducing a flare-up effect. 

**Figure 1:**
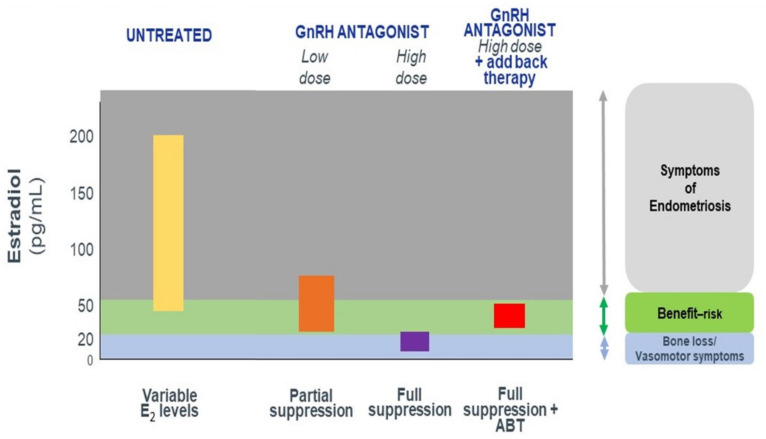
Expected E2 levels during the menstrual cycle and under gonadotropin-releasing hormone (GnRH) antagonist therapy with and without add-back therapy (ABT).

The main advantages of GnRH antagonists [13,21] are:

1. Oral administration.
2. Immediate suppression of FSH and LH secretion.
3. Dose-dependent estrogen suppression, from partial suppression at lower doses to full suppression at higher doses (Figure 1), suggesting the possibility of individual tailoring according to the symptoms and wishes of the patient.
4. Rapid reversibility and recovery of hormone secretion after stopping treatment.

Two oral GnRH antagonists (elagolix [44,45,46,47,48,49,50,51,52,53] and relugolix [54,55,56,57]) have already been approved by the American Food and Drug Administration (FDA) [44,55], and a third to emerge is linzagolix [58,59,60,61,62,63]. These three drugs have recently yielded very robust results in randomized, placebo-controlled clinical trials for the treatment of pain associated with endometriosis in symptomatic premenopausal women.

### 4.1. Elagolix

**Figure 2:**
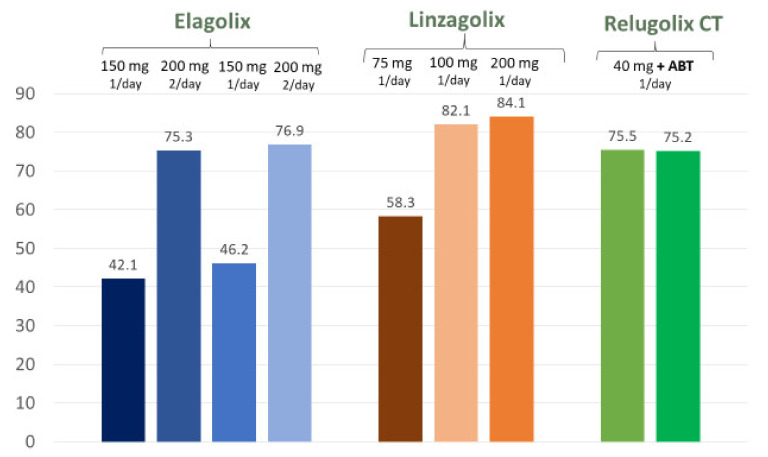
Percentage of subjects who showed clinically meaningful responses with respect to dysmenorrhea at 24 weeks.

The mean plasma half-life (t_1/2_) of elagolix ranges from 2.4 to 6.3 h [45,48,49]. The efficacy of 6 months of treatment with elagolix was evaluated in two large, double-blind, phase III trials (Elaris EM-I and Elaris EM-II) [48]. Two different regimens of elagolix (150 mg once daily and 200 mg twice daily) were tested. Two co-primary efficacy endpoints were the proportion of women who showed clinically meaningful responses with respect to dysmenorrhea, and non-menstrual pelvic pain at 3 months of treatment. In these EM-I and EM-II trials, the percentage of subjects who experienced a clinical improvement in dysmenorrhea at 12 weeks of treatment was 46.4% and 43.4% with 150 mg elagolix once daily, and 75.8% and 72.4% with 200 mg elagolix twice daily. The percentage of subjects who noted an improvement in non-menstrual pelvic pain was 50.4% and 49.8% with 150 mg elagolix once daily, and 54.5% and 57.8% with 200 mg elagolix twice daily (Table 1). Overall, alleviation of both dysmenorrhea and non-menstrual pelvic pain were sustained for 24 (Figure 2) and 52 weeks (Table 1) [48,49,50].

In phase III extension studies (Elaris EM-III and EM IV), subjects continued to receive elagolix for 6 additional months, with post-treatment follow-up of up to 12 months [49]. Upon completion of treatment, respective responder rates for dysmenorrhea in EM-III and EM-IV were 52.1% and 50.8% with 150 mg elagolix once daily, and 78.1% and 75.9% with 200 mg twice daily (Table 1). Responder rates for non-menstrual pelvic pain were 67.8% and 66.4% with 150 mg elagolix once daily, and 69.1% and 67.2% with 200 mg twice daily. At week 52, elagolix was found to cause a dose-dependent decrease in BMD (more than 3.5% at a dose of 200 mg) [47,48,49] versus 1% at a dose of 150 mg.

**Figure 3:**
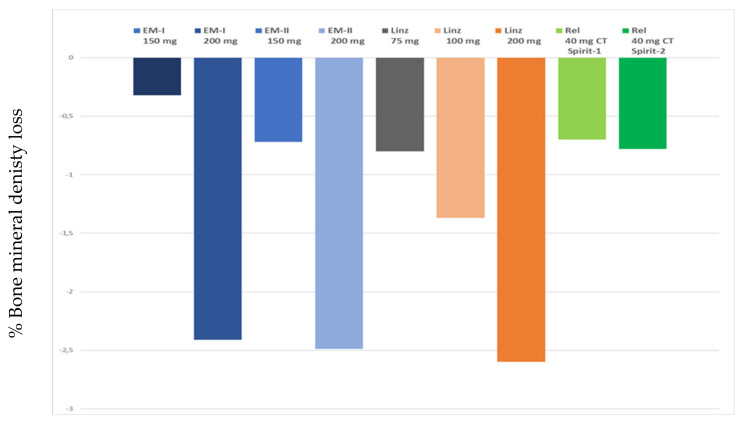
Mean percentage BMD loss at week 24 (lumbar spine) in women treated with different doses of gonadotropin-releasing hormone (GnRH) antagonist (150 mg elagolix once daily, 200 mg elagolix twice daily; 75 mg, 100 mg and 200 mg linzagolix once daily; and 40 mg relugolix plus ABT once daily).

In conclusion, use of 200 mg elagolix twice daily causes strong suppression of E2 and marked improvements in dysmenorrhea, non-menstrual pelvic pain, and dyspareunia, albeit at the cost of more hot flushes and a more pronounced decrease in BMD (Figure 3). Current research is focused on determining the impact of ABT during elagolix treatment [50], several papers reporting enhanced health-related quality of life in endometriosis patients undergoing this therapy [50,51,52,53].

### 4.2. Linzagolix

Linzagolix has a half-life of 15–18 h [58,59]. In a recent paper, Donnez et al. [58] evaluated the impact of linzagolix, a new oral GnRH antagonist administered once daily for 24 weeks, focusing on three doses that will be promoted by the company (75 mg, 100 mg, and 200 mg). Doses of 75 mg without ABT and 200 mg with ABT are currently being investigated in phase III endometriosis clinical trials.

Percentages of women experiencing a reduction of ≥30% in both dysmenorrhea and non-menstrual pelvic pain by 12 weeks of linzagolix treatment were respectively 68.2% and 58.5% in the 75 mg group, 68.6% and 61.5% in the 100 mg group, and 78.9% and 47.7% in the 200 mg group. Response rates for dysmenorrhea and non-menstrual pelvic pain were maintained or increased after 24 weeks of treatment (Table 1). Rates of those experiencing a reduction of >30% in both dysmenorrhea and non-menstrual pelvic pain by week 24 were 58.3% and 72.9% in the 75 mg group, 82.1% and 64.1% in the 100 mg group, and 84.1% and 72.7% in the 200 mg group.

Patients randomized to 200 mg linzagolix were switched to 100 mg linzagolix at week 24. Response rates for dysmenorrhea and non-menstrual pelvic pain were maintained or increased after 52 weeks of treatment [21]. Indeed, percentages of women experiencing a reduction of ≥30% in both dysmenorrhea and non-menstrual pelvic pain by week 52 were respectively 69.2% and 69.2% in the 75 mg group, 69.2% and 53.8% in the 100 mg group, and 64.7% and 76.5% in the 200/100 mg group. Based on EHP-30 questionnaire results, treatment with linzagolix resulted in enhanced quality of life. The EHP-30 questionnaire revealed pain and powerlessness domains to be significantly transformed with all three doses (75 mg, 100 mg, and 200 mg).

Concerning serum E2 levels, there was rapid and full suppression to 11 pg/mL, which was achieved in the 200 mg group by week 4 and maintained (range 11–16 pg/mL) to week 24. There was a dose-dependent partial suppression of serum E2 to between 20 and 60 pg/mL with 75 mg and 100 mg linzagolix. Amenorrhea rates from weeks 4 to 24 were also dose-dependent, yielding percentages of 36.3%, 55.8%, and 80.9% in the 75 mg, 100 mg, and 200 mg dose groups respectively. Hot flushes were more frequent in the 200 mg group.

Mean percentage BMD changes for the lumbar spine from baseline to week 24 in the 75 mg, 100 mg, and 200 mg groups were −0.80%, −1.37%, and −2.60% respectively (Figure 3). Subjects taking the 200 mg dose showed a BMD decrease that would require hormone ABT for longer-term use. It should be noted that calcium and vitamin D supplementation were not provided as part of the trial protocol [21,58]. At week 52, mean percentage BMD changes in the lumbar spine were −1.14 at a dose of 75 mg, −1.40 at a dose of 100 mg, and −2.19 at a dose of 200/100mg [61]. Hot flushes were more frequent at higher doses of linzagolix [21,61].

In conclusion, consistent with full suppression of serum E2 to postmenopausal levels, once daily 200 mg linzagolix has an additional significant impact on dyspareunia and some aspects of quality of life, as reported by Donnez et al. [58]. However, higher rates of hypoestrogenic symptoms were observed, including BMD loss of ≥3% in some women after 24 weeks, indicating that this once-daily dose will require hormone ABT if used for longer than 6 months.

### 4.3. Relugolix

**Figure 4:**
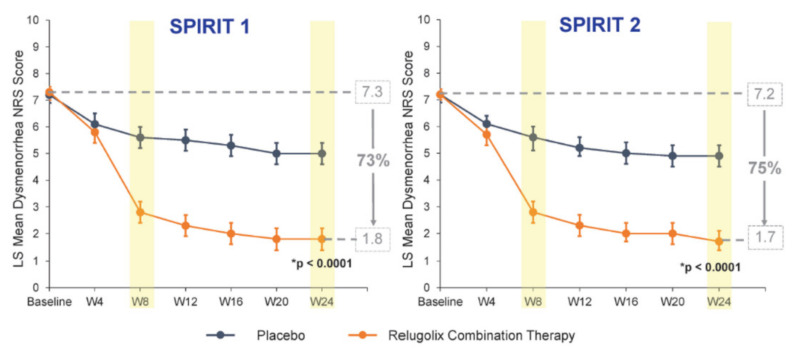
Dysmenorrhea rapidly decreased from severe at baseline to mild by week 8 and was sustained through to week 24. Error bars show the upper and lower limit of 95% confidence intervals; * *p* values compare relugolix combination therapy vs a placebo with respect to change in NRS dysmenorrhea defined previous page scores by week 24. CT = combination therapy; LS = least squares.

Relugolix has a half-life of 37–42 h [54,55]. Results of phase III clinical trials investigating the effects of relugolix on endometriosis (SPIRIT-1 and 2) were recently published [56,57] (Table 1). The drug was administered at a dose of 40 mg with ABT (1 mg E2 and 0.5 mg NETA). Percentages of women showing a mean reduction of ≥2.8 points in their numerical rating scale (NRS) scores for dysmenorrhea at week 24 were respectively 75.5% and 75.2% in SPIRIT-1 and 2. Dysmenorrhea decreased rapidly from severe at baseline to mild by week 8 and was sustained through to week 24 (Figure 4).

Percentages of women showing a mean reduction of ≥2.1 points for non-menstrual pelvic pain were 58.5% and 66%. Relugolix combination therapy (CT) enhanced daily functioning, as demonstrated by the Endometriosis Health Profile-30 (EHP-30) pain score domain at week 24 (*p* < 0.0001 in both studies). Mean percentage BMD changes in the lumbar spine from baseline to week 24 in the relugolix CT group were −0.70% in SPIRIT-1 and −0.78% in SPIRIT-2. Mean percentage hot flushes in the relugolix CT group at week 24 were 10.4% in SPIRIT-1 and 13.6% in SPIRIT-2 [56,57].

In conclusion, oral relugolix CT taken once daily significantly reduced dysmenorrhea and non-menstrual pelvic pain in women with endometriosis. Relugolix CT is well tolerated, the incidence of vasomotor symptoms is similar to a placebo, and BMD is maintained for 24 weeks.

## 5. Discussion and Conclusion: A Combined Symptom-Oriented and Phenotype-Adapted Approach

According to the ASRM Practice Committee [64], endometriosis requires a life-long management plan, with the goal of maximizing use of medical therapy and avoiding repeated surgical procedures. Following the first publication in the NEJM by Taylor et al. [48] on the impact of elagolix on endometriosis-associated pain and approval from the FDA [44], Vercellini et al. [65] concluded, in a paper entitled ‘All that glitters is not gold’, that the efficacy of GnRH antagonist should first be proved by pragmatic trials comparing elagolix with low-dose hormone contraceptives and progestogens. The authors argued that trade-offs between health outcomes and costs need to be carefully weighed up and proposed in a stepwise approach, starting with OCPs or low-cost progestogens and resorting to high-cost drugs only in case of inefficacy or intolerance [9,12,16]. However, a question needs to be asked: Why step up to high-cost drugs like dienogest, when these same authors failed to observe any significant differences in efficacy in the management of endometriosis-associated pain between NETA and dienogest [17]?

Moreover, in a recent paper [21], we thoroughly scrutinized the concept of progesterone resistance as an explanation for why 33% of patients do not respond to OCPs and progestins, with this percentage climbing even higher in women with deep nodular endometriosis [19,21,66,67,68]. As stated earlier, there is a need for further treatment options and a number of papers have reported results from clinical trials on three potentially useful oral GnRH antagonists: elagolix, linzagolix, and relugolix [44,45,46,47,48,49,50,51,52,53,54,55,56,57,58,59,60,61,62,63]. These studies clearly confirmed that GnRH antagonist suppresses ovarian function in a dose-dependent manner, allowing modulation of E2 levels which, according to the threshold hypothesis [5], may provide relief from endometriosis-associated pain while reducing side effects caused by extreme hypoestrogenism. Therefore, instead of the stepwise approach suggested by Vercellini et al. [65], we would prefer a strategy based on the main symptoms (pain and/or infertility) and the different phenotypes of endometriosis, clearly categorized into three separate entities in the original publication by Nisolle and Donnez in 1997 [2]. This would allow us to discriminate between lesions not only from a pathological and pathogenic point of view, but also from a clinical perspective [21].

### 5.1. Peritoneal Lesions

Laparoscopy remains the gold standard for detection of superficial implants and adhesions but, like any surgical intervention, it comes at a cost. It is well known that invisible lesions are present in at least 12% of cases [69], and recurrence is not an uncommon finding, obviously depending on the surgeon’s experience [70,71,72]. In the opinion of the authors, a bimanual examination and the Biberoglu and Behrman scale [73] remain important diagnostic tools, as they are easy to implement and allow the gynecologist to determine whether additional checks are required. In the vast majority of cases, transvaginal ultrasound will let the gynecologist exclude the presence of ovarian endometriomas, while bimanual examination can identify deep lesions.

**Figure 5:**
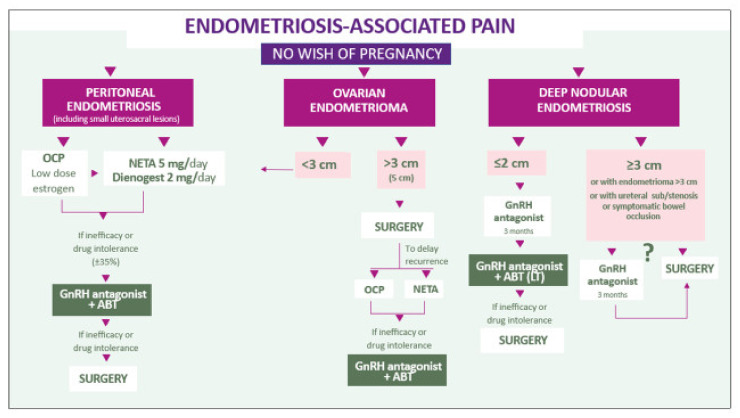
Combined symptom-oriented and phenotype-adapted approach. When the main symptom is endometriosis-associated pain and pregnancy is not desired, different algorithms are proposed according to the phenotype.

**Figure 6:**
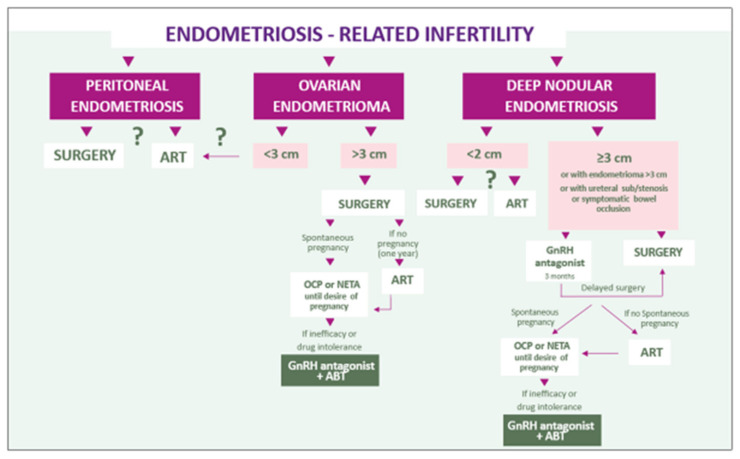
Combined symptom-oriented and phenotype-adapted approach to endometriosis-associated infertility.

Age and the wish to conceive will then influence the therapeutic decision (Figure 5 and Figure 6). As a first option, in the absence of nodular and/or painful uterosacral ligaments, OCPs or progestogens should be considered, especially if contraception is required. However, knowing that 33% of women are poor responders and that some patients will experience drug intolerance, other treatment options like GnRH antagonists with or without ABT should be contemplated [21,74]. Flores et al. [19] observed a correlation between the presence of PRs in endometriotic lesions and the response to estroprogestins and progestins. Although it is interesting to understand and confirm progesterone resistance a posteriori, taking a biopsy before starting therapy is clearly not recommended. Nevertheless, if for some other reason laparoscopy is required and reveals endometriotic lesions, biopsy and PR content evaluation may prove helpful to determine the appropriate therapy.

If bimanual pelvic examination identifies nodular uterosacral ligaments and symptoms include moderate-to-severe dysmenorrhea and dyspareunia, laparoscopy and endometriotic lesion excision may be proposed after explaining the pros and cons of surgery versus the medical approach to the patient. Physicians may be tempted to base their decision on their own values and competences, but this would be ill-advised. Indeed, there is growing agreement that greater emphasis should be placed on freedom to choose. Playing an active role in the decision-making process is now emerging as a favorable goal in itself in healthcare.

Vercellini et al. [7,9,12,65] proposed a kind of therapeutic pyramid, with a broad base of users of first-line medications (OCPs), a progressively narrower body of users of second-line drugs (progestins), and even narrower for patients using third-line therapies (GnRH agonist/antagonist), with a small number of patients undergoing surgery at the pyramid peak.

While we understand the concept of ‘stepping up’, we do not fully agree with the structure of the pyramid, with first-line, second-line, and third-line treatments administered before surgery, as suggested by Vercellini et al. [10,12,65]. Indeed, it is well known in clinical practice that patients easily lose confidence if different drugs are used repeatedly with poor results. In cases of moderate symptoms, we fully support the use of first-line therapy (OCPs or progestins) depending on the patient’s wish to menstruate or not. However, in the case of inefficacy or drug intolerance, use of an oral GnRH antagonist should at least be discussed and considered, as these drugs have proved to be effective at alleviating overall pelvic pain, as well as dysmenorrhea and non-menstrual pelvic pain. Future studies evaluating the efficacy of GnRh antagonists in case of inefficacy of OCPs or progestins are needed to confirm the validity of this approach.

### 5.2. Endometriomas

The debate around the best approach in case of endometriomas is now a hot topic and more specifically related to endometrioma-associated infertility. A recent ‘Fertile Battle’ [75] reports that women with endometrioma-related infertility face a dilemma when choosing appropriate therapy: surgery or in vitro fertilization [76]. In fact, ovarian endometriomas respond poorly to medical therapy (OCPs progestins and even GnRH agonist) essentially due to their anatomical structure, which is a fibrotic pseudocapsule surrounding chocolate fluid that constitutes 95% of the content [2,77,78]. Frequent inflammation of the ovarian stroma around the endometrioma, which is itself responsible for a depleted ovarian reserve [79,80], may represent one avenue of research into a medical approach to fertility preservation in the future.

According to European Society of Human Reproduction and Embryology (ESHRE) guidelines [81], the choice of surgery versus medical therapy depends on endometrioma size (at least 3 to 4 cm), the wish to conceive, any association with uterosacral or deep lesions, and of course patient age, the skill of the surgeon, and levels of ovarian reserve markers (anti-Müllerian hormone and antral follicle count) [82,83].

As some papers have reported lower recurrence rates after surgery when medical therapy was given postoperatively, [1,43,64,81,84,85], this option should be investigated by further clinical trials, bearing in mind that side effects should be kept to a minimum. In women undergoing surgery for endometriomas but not seeking immediate conception, advantages of long-term post-operative therapy are clear in terms of recurrence. Here again, the pros and cons of the different medical options should be discussed with the patient [76].

### 5.3. Deep Endometriosis

Deep nodular endometriotic lesions are often associated with severe pelvic pain. Their progression is slow [2,86,87] and it is difficult at the time of diagnosis to establish exactly when the lesion developed, grew, and ceased its evolution. Nevertheless, nobody is born with stage IV endometriosis [1] and we must acknowledge that the lesion was progressive at some point in time. However, the original definition of deep-infiltrating lesions as lesions invading the retroperitoneal space by >5 mm is now more than obsolete [86,87,88,89,90]. Deep endometriosis should be defined as nodules measuring at least 2–3 cm fixed to the posterior part of the cervix, most with posterior extension to the rectal muscularis [88,89,90].

Deep lesions are often hyperalgic because of peri- and intraneural invasion by endometriotic foci [91,92,93,94]. They are also associated with richly innervated areas [95,96] and their localization (at the level of the Douglas pouch) explains the dyspareunia experienced by patients and severe pain provoked by manual pelvic examination. The response of deep endometriotic nodules to medical therapy (progestins or OCPs) has long been a source of controversy. Some authors [8,17,97,98] observed a substantial volume reduction with OPCs and NETA therapy in favor of progestins, but this has not been confirmed in other more recent studies, where many authors found progestins to be relatively ineffective [68]. Indeed, a recent review by Reis et al. [68] also confirms that deep endometriosis looks to be more resistant to size regression upon medical treatment. Even if some PRs are present, they could be biologically inactive [1,2]. On the other hand, PRs may be absent, causing progesterone resistance and no cytoreduction of lesions [19,23,24,25,26,68,74,99].

Most guidelines consider that surgery plays an important role in the management of symptomatic deep endometriosis and recognize the benefits of surgery [64,81,85,100]. However, these patients need to be treated in referral centers of expertise, adopting a multidisciplinary approach, including urologists and a colorectal surgeon [88,90]. GnRH antagonist will likely reduce the size of deep lesions, similarly to GnRH agonist, or at least the induced hypoestrogenism will decrease the surrounding inflammation, vascularization, and infiltration, allowing less aggressive surgery than bowel resection. Bowel resection is known to have a high rate of complications compared to shaving [88,90], and efforts should be made to lower the rate of bowel resection, which in some countries exceeds 50%.

Further studies are needed to define the specific role of GnRH antagonist in the management of deep endometriosis, which the authors propose as preoperative therapy (Figure 6). However, in case of an excellent response, why not continue with GnRH antagonist and ABT to avoid surgery altogether? GnRH antagonist therapy should be systematically adopted in case of pain recurrence after surgery, as second surgery is associated with significantly more complications and fewer benefits in terms of pain [88,89,90,99,101,102].

One pilot study on a small number of patients (*n* = 10) who experienced recurrence of severe pelvic pain after one surgical procedure for deep endometriosis demonstrated the high efficacy of GnRH antagonist (cetrorelix depot, intramuscular, once a week) in terms of pain relief in this specific group [103]. Side effects were minimized, as E2 levels were maintained in the optimal range according to the threshold hypothesis [5]. This surely warrants further investigation into the benefits of long-term GnRH antagonist therapy in case of recurrence of severe pelvic pain after surgery for deep endometriosis, or in women who delay attempts to conceive [21].

## 6. Conclusions

Appropriate counseling of patients is of fundamental importance. It is the responsibility of healthcare workers to provide a comprehensive overview of the efficacy and side effects of all available therapies. The ideal treatment should be tailored to each and every woman according to the most distressing symptoms (pain and/or infertility) (Figure 5 and Figure 6) and the phenotype of the disease. Long-term adherence of patients to the treatment is crucial.

In this context, efficacy and side effects are key points to take into account. Indeed, the first goal of medical therapy is to be effective and avoid unnecessary surgical procedures. Even more importantly, it should prevent repeat surgery in case of recurrence of pain after surgery, as it is widely acknowledged that reoperation is often the source of severe complications.

We certainly cannot overlook the cost-effectiveness of medical management of endometriosis, but on the other hand, costs linked to endometriosis are already estimated at $69.4 billion per year [104,105]. It is time to promote research, encourage innovation in treatment options, and improve women’s access to quality care. Moreover, according to Wang et al. [106], two recently FDA-approved doses of elagolix for management of moderate-to-severe pain associated with endometriosis (24 months, 150 mg, once daily; and 6 months, 200 mg, twice daily) both proved cost-effective versus leuprolide acetate over a time frame of 1–2 years. Although there are still areas that require further scrutiny in terms of efficacy and safety in real-world populations, potential use of ABT, and comparisons with OCPs and progestins [107], we agree with Leyland et al. [108] that clinical evidence clearly demonstrates that oral GnRH antagonists are effective and well tolerated in patients with moderate-to-severe endometriosis-associated pain. Of course, studies comparing the efficacy of GnRH antagonists with OCPs and progestins are mandatory.

## Figures and Tables

**Table 1:** Efficacy of different doses of gonadotropin-releasing hormone (GnRH) antagonist at 24 and 52 weeks (150 mg elagolix once daily; 200 mg elagolix twice daily; 75 mg, 100 mg, and 200 mg linzagolix once daily; and 40 mg relugolix plus ABT once daily). NMPP: non-menstrual pelvic pain; * NA: not available. Patients randomized to linzagolix 200 mg were switched to 100 mg linzagolix at week 24).

Type of Drug	Elagolix				Linzagolix			Relugolix + ABT	
Dose	150 mg	200 mg (Twice Daily)	150 mg	200 mg (Twice Daily)	75 mg	100 mg	200 mg	40 mg + ABT	40 mg + ABT
**Assessments at Week 12**									
Dysmenorrhea (% responders)	46.4	75.8	43.4	72.4	68.2	68.6	68.9	NA	NA
NMPP (% responders)	50.4	54.5	49.8	57.8	58.5	61.5	47.7	NA	NA
**Assessments at Week 24**									
Dysmenorrhea (% responders)	42.1	75.3	46.2	76.9	58.3	82.1	84.1	75.5	75.2
NMPP (% responders)	45.7	62.1	51.6	62.2	72.9	64.1	72.7	58.5	66
**Assessments at Week 52**									
Dysmenorrhea (% responders)	52.1	78.1	50.8	75.9	69.2	69.2	64.7 *	NA	NA
NMPP (% responders)	67.8	69.1	66.4	67.2	69.2	53.8	76.5 *	NA	NA
